# Incorporating the Soil Gas Gradient Method and Functional Genes to Assess the Natural Source Zone Depletion at a Petroleum-Hydrocarbon-Contaminated Site of a Purification Plant in Northwest China

**DOI:** 10.3390/life13010114

**Published:** 2022-12-30

**Authors:** Zhuo Ning, Yizhi Sheng, Caijuan Guo, Shuaiwei Wang, Shuai Yang, Min Zhang

**Affiliations:** 1 Institute of Hydrogeology and Environmental Geology, Chinese Academy of Geological Sciences, Shijiazhuang 050061, China; 2 Key Laboratory of Groundwater Remediation of Hebei Province & China Geological Survey, Shijiazhuang 050061, China; 3 Center for Geomicrobiology and Biogeochemistry Research, State Key Laboratory of Biogeology and Environmental Geology, China University of Geosciences, Beijing 100083, China; 4 SINOPEC Research Institute of Safety Engineering Co., Ltd., Qingdao 266000, China

**Keywords:** natural source zone depletion (NSZD), petroleum hydrocarbon contaminated site, gradient method, functional gene

## Abstract

An increasing number of studies have demonstrated that natural source zone depletion (NSZD) in the vadose zone accounts for the majority (90%~99%) of the natural attenuation of light non-aqueous phase liquid (LNAPL). Until now, 0.05 to 12 kg/a.m^2^ NSZD rates at tens of petroleum LNAPL source zones have been determined in the middle or late evolution stage of LNAPL release, in which limited volatile organic compounds (VOCs) and methane (CH_4_) were detected. NSZD rates are normally estimated by the gradient method, yet the associated functional microbial activity remains poorly investigated. Herein, the NSZD at an LNAPL-releasing site was studied using both soil gas gradient methods quantifying the O_2_, CO_2_, CH_4_, and VOCs concentrations and molecular biology methods quantifying the abundance of the *pmo*A and *mcr*A genes. The results showed that the methanogenesis rates were around 4 to 40 kg/a.m^2^. The values were greater than the rates calculated by the sum of CH_4_ escaping (0.3~1.2 kg/a.m^2^) and O_2_ consuming (3~13 kg/a.m^2^) or CO_2_ generating rates (2~4 kg/a.m^2^), suggesting that the generated CH_4_ was oxidized but not thoroughly to CO_2_. The functional gene quantification also supported the indication of this process. Therefore, the NSZD rates at the site roughly equaled the methanogenesis rates (4~40 kg/a.m^2^), which were greater than most of the previously studied sites with a 90th percentile value of 4 kg/a.m^2^. The study extended the current knowledge of the NSZD and has significant implications for LNAPL remediation management.

## 1. Introduction

Natural source zone depletion (NSZD) is the natural loss of light non-aqueous phase liquid (LNAPL) through collective, naturally occurring processes of volatilization, dissolution, and biodegradation [1,2,3]. A growing number of studies have demonstrated that NSZD occurs at petroleum-hydrocarbon-contaminated sites at depletion rates ranging from thousands to tens of thousands of liters per hectare per year. Approximately 90% to 99% of the natural attenuation of petroleum hydrocarbons occurs in the vadose zone, the area extending from the surface to the regional groundwater table [4,5]. Across the petroleum-hydrocarbon-contaminated aquifer to the vadose zone, three zones, i.e., the methane generation zone, methane oxidation zone, and aerobic transport zone are defined by the one-dimensional conceptual model in terms of their key roles in NSZD [6]. Methanogenesis occurs in the saturated aquifer and deep vadose zone (e.g., capillary zone), where the biodegradation of LNAPL compounds is accomplished by a syntrophy between fermenters and methanogens to produce methane (CH_4_) and carbon dioxide (CO_2_). Non-syntrophic methanogenic hydrocarbon degradation by an archaeal species has also been found [7]. Through ebullition and diffusion, the generated gases migrate toward the surface. In the vadose zone, aerobic bacteria convert a proportion of CH_4_ into CO_2_, which diffuses upward to the surface of the ground.

It is currently possible to determine the NSZD rate using several methods, including gradients, carbon traps, dynamic closed chambers, biogenic heat, and the recently proposed compositional change method for LNAPL, of which the first four methods are the most commonly used [8,9]. For each of the four methods, more than ten sites have been studied [9]. The gradient method is the earliest and most well-studied, which was systematically proposed by Johnson et al. [10]. With the help of measurements of the changes in soil gas concentrations and respective stoichiometric relationships, NSZD rates can be readily estimated. The soil-gas constituent concentration gradients are obtained by assessing the changes in the vertical distribution of soil gas constituents (O_2_, CO_2_, CH_4_, and vapor-phase petroleum hydrocarbon) in the vadose zone above the LNAPL source. Combined with the estimated or field-measured soil gas effective diffusion coefficient, the gaseous mass flux is calculated using Fick’s first law of diffusion. Stoichiometric conversion of the biodegradation product mass flux then allows an estimate of the rate of LNAPL mass depletion [8]. The gradient method gives instantaneous measurement and facilitates the identification of distinct physicochemical pathways in NSZD [11]. Owing to its advantages, this method has been universally applied to estimate NSZD under various conditions [5]. The method was first applied in an aged oil field [8,12]. Gradient methods were then used to estimate NSZD rates in various LNAPL-contaminated sites, including crude oil, diesel, jet fuel, and gas condensate, etc. [4,13,14,15,16,17].

A majority of the studies were conducted in closed sites at mid- or late-stages in LNAPL release [14,18], during which fewer volatile petroleum hydrocarbons and CH_4_ were detected in the vadose zone. As such, volatile petroleum hydrocarbons and CH_4_ were always excluded or omitted when calculating NSZD rates [19]—whereas in the early evolutionary stage of LNAPL-releasing sites, CH_4_ and VOCs should be taken into account [4]. Moreover, microbial activities during NSZD have rarely been investigated.

In the study, therefore, NSZD at a LNAPL-releasing site of a purification plant in Northwest China was assessed by the gradient method coupled with the quantification of the core genes encoding the methanogenesis and methane oxidation processes. This study extended for the first time the current knowledge of NSZD assessment in China, which is broadly applicable to thousands of similar petroleum-hydrocarbon-contaminated sites [20].

## 2. Materials and Methods

### 2.1. Site Description

**Figure 1:**
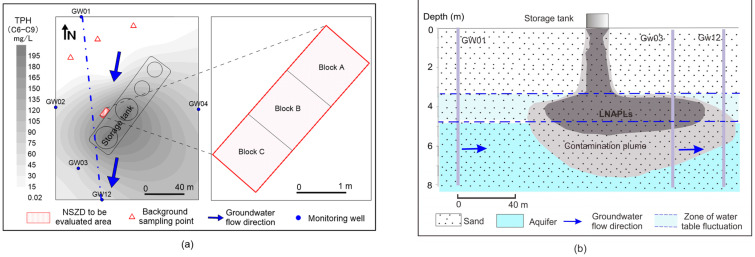
The characteristics of the study area and LNAPL contamination. Plot (**a**) shows the sampling location, groundwater flow direction, and the TPH contaminant concentrations (as represented by the concentration contour plot). The red rectangle near the storage tanks (i.e., block A, B, and C) represents the area where NSZD was evaluated. The three red triangles in the non-contaminated area, north of the storage tanks, represent the background sampling points. Plot (**b**) shows the vertical distribution of the LNAPLs and contamination plume.

The study site is located near the gas condensate storage tanks at a purification plant in Northwest China. The depth of the water table at the site ranges from 3.2 to 4.7m below the ground surface. The vadose zone and saturated zone are primarily composed of fine sands. The groundwater flow direction is generally from north to south. Due to the gas condensate released from the storage tanks and the impact of groundwater flow, the contaminants spread nearly all over the site (Figure 1a). LNAPL was mainly present in the zone of water table fluctuation (Figure 1b). Contaminants at the site were largely occupied by the light-end petroleum hydrocarbons such as benzenes and other smaller molecule volatile petroleum hydrocarbons (C_6_–C_9_).

### 2.2. Sampling, Gas Measurement, and Functional Gene Determination

The most likely contamination source zone to the north of the in-service storage tanks was chosen to assess the rate of natural source zone depletion. Regarding the complex pipeline underground and the presence of flammable and explosive hydrocarbons, it was a forbidden area for traditional invasive drilling and long-term retention of monitoring wells according to the strict rules of the plant. To gain the NSZD rate and data on the different physicochemical pathways of NSZD, a minimally invasive measurement was carried out in the study area. The area was divided into 3 one-meter square blocks (Figure 1a). All blocks were treated as replicates. The concentrations of soil gases and functional genes were measured in each block. Meanwhile, the non-contaminated area was chosen as the background.

(1) Soil gas concentration measurement

Using a 2-cm-diameter, 1.0-m-long copper drill rod hammered with a copper hammer, boreholes with different depths were drilled. First, a 10-cm-deep hole was drilled. The concentrations of VOCs, CO_2_, O_2_, H_2_, and CH_4_ were immediately measured in the drilled hole using a portable multi-parameter gas detector (MultiRAE 6208, USA). Afterward, in the same way, the hole was deepened to 20 cm and the gas concentrations were measured. The same procedure was applied to a depth of 90 cm. Triplicate measurements were conducted in each block.

Due to the fact that the gas measured in the borehole was a mixture of gas extracted from different soil depths, the measured concentrations in each layer might not fully represent their real concentrations. In the current study, to obtain the real concentration in each layer, we proposed a procedure to estimate the real gas concentrations in a certain depth layer.

The assumptions of the measurement were (i) the soil column is homogeneous within 90 cm, i.e., the gas permeabilities are uniform; (ii) at the same depth, the gas concentrations are the same anywhere; (iii) during the course of gas extraction and measurement, the gas is equally extracted from different depths, i.e., soil gases from different depths have the same contribution; (iv) when the measured gas concentration values are stabilized, the stabilized values represent the soil gas concentrations.

**Figure 2:**
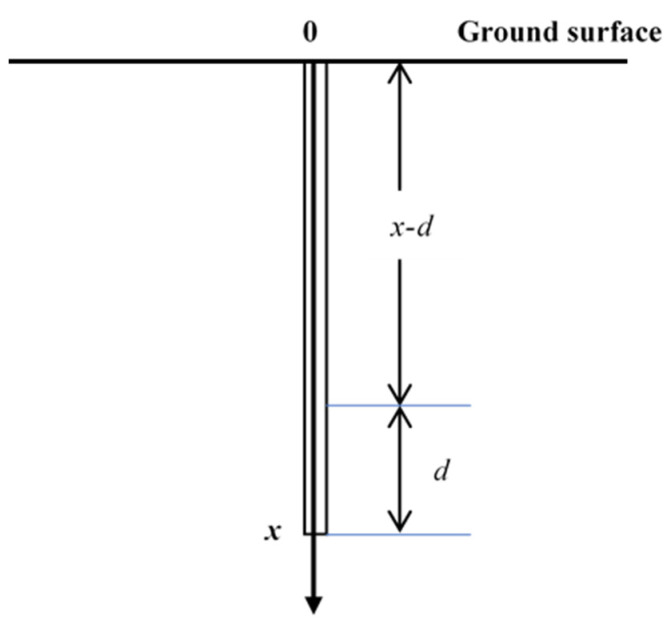
Schematic plot of the soil gas measurement borehole.

Then, the amount of a certain gas in the layer between (*x − d*) and *x* depths (Figure 2) can be expressed as

(1) $$M\left(x-d,x\right)=\int_{x-d}^{x}C\left(x\right)dx$$

where *x* is the depth below the ground surface, m;

*C* (*x*) is the actual gas concentration at *x* depth, g/m^3^;

*M* (*x − d, x*) is the amount of the gas in the layer between *x − d* and *x* depths, g/m^2^.

Assuming the measured concentration in the *x*-depth hole is *C_m_*(*x*), the amount of the gas in the hole can be expressed as

(2) $$M\left(0,x\right)=C_{m}\left(x\right)\cdot x$$

The amount of the gas in the *x*-depth hole can be deemed as the amount of the gas in the *x − d* depth hole plus the amount of the gas in the layer between the *x − d* and *x* depths, and can therefore be expressed as

(3) $$\begin{array}{c} M\left(0,x\right)=C_{m}(x-d)\cdot (x-d)+M\left(x-d,x\right)\\ \;\;\;\;\;\;\;\;\;\;\;\;=C_{m}(x-d)\cdot (x-d)+\int_{x-d}^{x}C\left(x\right)dx \end{array}$$

Therefore,

(4) $$C_{m}(x)\cdot x=C_{m}(x-d)\cdot (x-d)+\int_{x-d}^{x}C\left(x\right)dx$$

For assessing NSZD in practice, when *d* is small enough, between *x − d* and *x* depths, *C_a_*(*x*) can be deemed as a constant value. Then, Equation (4) can be transformed as

(5) $$C_{m}(x)\cdot x=C_{m}(x-d)\cdot (x-d)+C\left(x\right)\cdot d$$

*C_m_*(*x*) and *C_m_*(*x − d*) in Equation (5) are easily measured. Then, the actual gas concentration at *x* depth can be calculated as

(6) $$C\left(x\right)=\frac{C_{m}\left(x\right)\cdot x-C_{m}(x-d)\cdot (x-d)}{d}$$

In the present study, *d* was set as 0.1 m.

(2) Functional gene determination

To verify the crucial biochemical processes upon NSZD, e.g., methanogenesis and methane oxidation, the corresponding functional genes methyl coenzyme M reductase gene (*mcrA*) and methane monooxygenase gene (*pmoA*) were quantified. The detailed procedure was as follows: At each depth of the gas concentration measurement, an approximately 50 g soil sample was collected using the Luoyang shovel and sealed in a sterile bag. Then, the soil samples were stored in an incubator filled with dry ice and transported to the laboratory for DNA extraction. The DNA was extracted from 0.8 g soil of each sample using a FastDNA kit (Q-BIO gene Corp. Irvine, CA, USA) [21]. Then, the *mcrA* and *pmoA* gene abundance was quantified using a fluorescence quantitative PCR instrument (ABI Q5, USA). The PCR primers and detailed experimental procedures are available in the relevant references [22,23].

### 2.3. Assessment of the Natural Source Zone Depletion

After the *C*(*x*) values of O_2_, CH_4_, and VOCs were gained, the NSZD rate could be calculated according to Equation [19]

(7) $$R=-D_{\mathrm{HC}}\frac{\partial C_{\mathrm{HC}}(x)}{\partial x}-S_{{\mathrm{CH}}_{4}}\cdot D_{{\mathrm{CH}}_{4}}\frac{\partial C_{{\mathrm{CH}}_{4}}(x)}{\partial x}+S_{O_{2}}\cdot D_{O_{2}}\frac{\partial C_{O_{2}}(x)}{\partial x}$$

where $\frac{\partial C_{\mathrm{HC}}(x)}{\partial x}$, $\frac{\partial C_{\mathrm{CH}}{}_{{}_{{}_{4}}}(x)}{\partial x}$, $\frac{\partial C_{O_{2}}(x)}{\partial x}$ represent the vertical concentration gradients of hydrocarbon, methane, and oxygen, respectively, at depth (*x*) of the horizontal plane in (g/m^3^)/m;

*S*_CH4_ is the stoichiometric coefficient for methanogenesis, and the value is 1.1 g-HC/g-CH_4_;

*S*_O2_ is the stoichiometric coefficient for aerobic biodegradation, ranging from approximately 0.25 to 0.29 g hydrocarbon/g O_2_ consumed, depending on the relative contributions of direct hydrocarbon aerobic oxidation (0.29 kg hydrocarbon/mg O_2_) and indirect hydrocarbon oxidation (0.25 mg hydrocarbon/mg O_2_, assuming that methane production occurs first in the anaerobic source zone and then methane is subsequently biodegraded aerobically as it diffuses upward);

*D*_HC_, *D*_CH4_, and *D*_O2_ represent the effective vapor phase diffusion coefficients for hydrocarbon, methane, and oxygen, respectively, at depth (*x*) of the horizontal plane in m^2^/s.

Effective diffusion coefficients were estimated by the most widely used Penman model [24].

(8) $$D=0.66\varepsilon \cdot D_{0}$$

where *D* is the gas diffusion coefficient in soil (m^2^/s);

*D*_0_ is the gas diffusion coefficient in free air (m^2^/s);

*ε* is the soil air-filled porosity.

According to Equation (8), the effective vapor phase diffusion coefficients for hydrocarbon (represented by benzene), CH_4_, O_2_, and CO_2_ were calculated to be 1.27 × 10^−4^ m^2^/s, 3.08 × 10^−5^ m^2^/s, 2.47 × 10^−5^ m^2^/s, and 2.16 × 10^−5^ m^2^/s respectively. In the estimation, the air-filled porosity was measured to be 0.2 and the gas diffusion coefficients in free air were gained from the work of Massman [25].

In this study, the CO_2_ and CH_4_ gradients were also used to assess the CO_2_ production rate and methanogenesis rate, respectively.

## 3. Results and Discussion

### 3.1. Soil Gas Profiles

**Figure 3:**
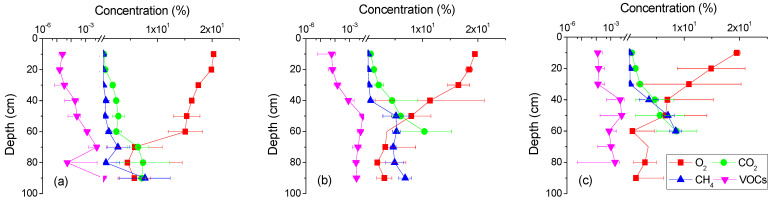
Vertical profile of soil gas O_2_, CO_2_, CH_4_, and VOCs calculated in the study area. Some missed data points at 70~90cm indicated that the concentrations were greater than the upper limits of detection. (**a**–**c**) Plots represent the A, B, and C blocks in the contamination source area, respectively.

Based on the minimally invasive measurement of soil gas concentrations, the actual soil gas concentrations in the study area were calculated using Equation (6), and they are shown in Figure 3. It can be observed that the O_2_ concentrations rapidly decreased with depth above 60 cm in all blocks, while the VOCs, CO_2_, and CH_4_ concentrations increased. Below 60 cm depth, the VOCs and O_2_ concentrations did not vary with depth and exhibited some fluctuation, while the CO_2_ and CH_4_ concentrations continued to increase with depth in blocks A and B, and were greater than the upper limits of detection in block C.

Although the VOCs were detected and basically increased with depth in all blocks, the concentrations of VOCs are about 2 to 4 orders lower than CO_2_ and CH_4_. The trend of CO_2_ concentration variations and O_2_ displayed a significant negative correlation (r = −0.967, *p* < 0.001). The depth-dependent variations in CH_4_ concentration were nonlinear and had different patterns. Above the 50cm, 40cm, and 30cm for blocks A, B, and C, the CH_4_ concentration was less than 1% with fewer variations, while below these depths, the CH_4_ concentration was greater than 1% and almost linearly increased with depth. The lower CH_4_ concentration in the upper layers (<50 cm, 40 cm, and 30 cm for blocks A, B, and C) indicated that there might be less methanogenesis and more methane oxidation, and the O_2_ concentration gradients were mainly formed by diffusion.

### 3.2. Soil Gas Gradients

To calculate the NSZD rate, only the O_2_ concentrations in diffusion-dominated areas were taken into consideration. As methane oxidation also generates CO_2_, the CO_2_ diffusion-dominant areas were the same as for O_2_. It was hard to distinguish the CH_4_ and VOCs diffusion-dominated areas because CH_4_ and VOCs might be oxidized in the space coexisting with O_2_. If the oxidation rates were constant at different depths, the concentration gradients would be only caused by diffusion. Upon this assumption, to estimate the rates of VOCs volatilization, the concentrations at the same depths as O_2_ diffusion-dominated depths were taken into account; and to estimate the rates of CH_4_ generation in the source zone, the concentrations in the soil below the mutational depths (>50 cm, 40 cm, and 30 cm for blocks A, B, and C) were taken into consideration. Herein, the linear curves were fitted with the gas profile data. The fitted depths, calculated gradient, and adj. R^2^ are listed in Table 1.

As shown by the adj. R^2^ values in Table 1, the concentrations of O_2_, CO_2_, and CH_4_ were well fitted, whereas the concentrations of VOCs were not.

### 3.3. Estimating the NSZD Rates

In agreement with the prior assumption, in the O_2_ diffusion-dominated area, all soil gas concentrations only varied by diffusion, and the NSZD rate in each block could be calculated according to Equation (7). The amounts of O_2_ consumed hydrocarbons in blocks A, B, and C and the background were calculated to be 3.9 ± 0.4 kg/a.m^2^, 3.5 ± 0.7 kg/a.m^2^, 12.3 ± 0.4 kg/a.m^2^ and 0.1 ± 0.0 kg/a.m^2^, respectively. The amounts of CH_4_ escaping to the air corresponding to biodegraded hydrocarbons in blocks A, B, and C and the background were calculated to be 0.9 ± 0.3 kg/a.m^2^, 0.4 ± 0.2 kg/a.m^2^, 0.3 ± 0.0 kg/a.m^2^, and 0.0 kg/a.m^2^, respectively. The calculated VOCs volatilization rates were less than 0.001 kg/a.m^2^ in all areas. Therefore, the total NSZD rates were 4.7 ± 0.7 kg/a.m^2^, 3.4 ± 0.9 kg/a.m^2^, and 12.5 ± 0.4 kg/a.m^2^ in accordance with the O_2_ estimation.

In addition to the use of O_2_ consumption to estimate the NSZD, the product of hydrocarbon biodegradation, CO_2_, was also used to estimate the NSZD. The CO_2_ generation corresponding to NSZD fluxes was calculated to be 3.3 ± 0.4 kg/a.m^2^, 2.8 ± 0.5 kg/a.m^2^, 3.4 ± 0.4 kg/a.m^2^, and 0.1 ± 0.0 kg/a.m^2^ in blocks A, B, and C and the background, respectively. Accordingly, the total NSZD rates were 4.1 ± 0.7 kg/a.m^2^, 3.1 ± 0.7 kg/a.m^2^, and 3.7 ± 0.4 kg/a.m^2^ in accordance with the CO_2_ estimation.

Based on the CH_4_ concentration gradients in the deeper soils (>50 cm, 40 cm, and 30 cm for blocks A, B, and C), the methanogenesis-caused NSZD rates were calculated to be 6.7 ± 2.1 kg/a.m^2^, 9.3 ± 2.4 kg/a.m^2^, and 41.7 ± 1.7 kg/a.m^2^ in the A, B, and C blocks, respectively.

### 3.4. Functional Genes Evidence

**Figure 4:**
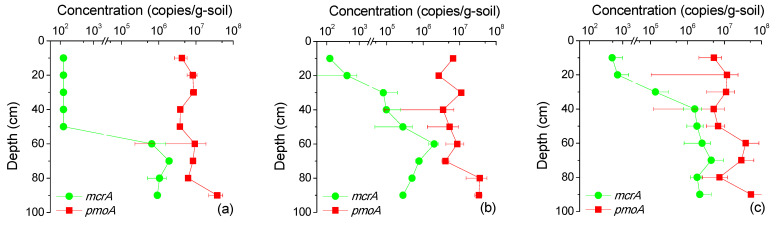
Vertical profile of *pmo*A and *mcr*A gene abundance in the study area. The (**a**–**c**) plots represent the A, B, and C blocks in the contamination source area, respectively.

The *mcr*A gene, encoding the methyl coenzyme M reductase (MCR) complex, remained at a low level in the upper layer until depths deeper than 60 cm, where it remained steady (Figure 4). This trend was the opposite of the depth-dependent oxygen variation. The correlation analysis results showed that *mcr*A gene concentrations had a significantly negative correlation with O_2_ concentrations (r = −0.740, *p* < 0.001) and a significantly positive correlation with CH_4_ concentrations (r = −0.686, *p* < 0.001), but were not significantly correlated with VOCs (r = 0.133, *p* = 0.518).

**Figure 5:**
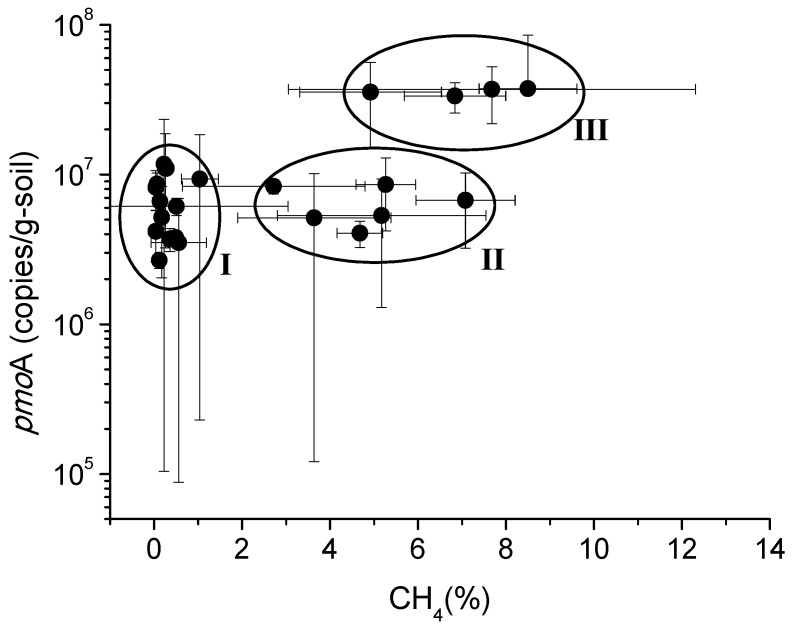
The relationships between *pmo*A gene abundance and CH_4_ concentration.

The concentration of the *pmo*A gene, encoding methane monooxygenase, did not vary as dramatically as the *mcr*A gene (Figure 4). The correlation analysis results showed that the *pmo*A gene had a significantly negative correlation with O_2_ (r = −0.526, *p* = 0.005) and a positive correlation with CH_4_ (r = 0.669, *p* < 0.001). As Figure 5 illustrates, CH_4_ and the *pmo*A gene were not linearly correlated. The relationships can be divided into three circumstances: (I) low CH_4_ and low-to-middle *pmo*A, corresponding to the samples collected from the surface or near the surface; (II) middle CH_4_ and middle *pmo*A, correspondng to the samples collected from the intermediate depth (about 30–80 cm); and (III) high CH_4_ and high *pmo*A, corresponding to the samples collected from the deep depth (greater than 60 cm). There was no significant difference between *pmo*A concentrations in group I and group II (*p* < 0.05), indicating that the CH_4_ oxidation rates in these areas were basically invariant, while the *pmo*A concentrations in group III were significantly higher. 

## 4. Discussion

### 4.1. Biogeochemical Process Process in NSZD

All A–C soil gas profiles displayed more apparent O_2_ usage and CO_2_ and CH_4_ production in the deeper subsurface. The concentrations of VOCs were about 2 to 4 orders lower than CO_2_ and CH_4_, which suggested that nonmethane hydrocarbon contaminants were scarcely depleted through volatilization, but were rather potentially biodegraded by functional microorganisms in the source zone, consistent with previous studies [4,13,14,15,16,17,19]. For the background (uncontaminated) samples, no VOCs and CH_4_ were generated. The calculated O_2_ usage and CO_2_ generation due to hydrocarbon depletion fluxes were both around 0.1 kg/a.m^2^, which may merely reflect the soil respiration rates [4] and only accounted for 1%~4% of the values in the contaminated samples (i.e., A, B, and C blocks). Taken together, natural depletion was in progress in the source zone.

Below the 50cm, 40cm, and 30cm depths for blocks A, B, and C, the CH_4_ concentrations were greater than 1% and almost linearly increased with depth. This result was not consistent with most of the sites, where CH_4_ was always undetected in the vadose zone [4,17,19]. The high concentrations of CH_4_ may have been caused by the continuous leaking of hydrocarbons in the in-service storage tanks, which supplied enough substrate for methanogenesis [4]. In anaerobic environments contaminated by LNAPLs, fermenters would biodegrade hydrocarbons and form dissolved hydrogen and/or acetate, which would be further utilized by methanogens as a substrate to form methane [26,27]. The lower CH_4_ concentrations in the upper layers (<50 cm, 40 cm, and 30 cm for block A, B, and C) indicated that there might be less methanogenesis and more methane oxidation, and the O_2_ concentration gradients were mainly formed by diffusion. In the deeper layers, O_2_ may also be consumed by methane oxidation. Taken together, the soil gas profiles in the study area were consistent with the NSZD model showing the presence of both aerobic and anaerobic biodegradation in the source zone of the site.

The CO_2_ estimated NSZD rates (2.4~4.8 kg/a.m^2^) were slightly lower than the O_2_ estimated values (2.5~12.9 kg/a.m^2^). CO_2_ is the ultimate product of hydrocarbon biodegradation; therefore, some intermediate products are excluded when estimating NSZD using CO_2_. While O_2_ is used, some hydrocarbons may not be thoroughly biodegraded to CO_2_ but are included in the NSZD calculation. This may result in lower estimated values of CO_2_. According to the NSZD concept model, as for O_2_-consuming processes in the vadose zone, only CH_4_ oxidation is taken into consideration. Therefore, it is essential to evaluate the methanogenesis fluxes.

The calculated NSZD rates caused by methanogenesis (4~40 kg/a.m^2^) were greater than the NSZD rates estimated by both O_2_ and CO_2_ gradients. One plausible reason is that the generated CH_4_ was not thoroughly oxidized to CO_2_. The stoichiometric relationships between O_2_ consuming (or CO_2_ generating) and hydrocarbon biodegrading processes in Equation (7) are based on the reaction that CH_4_ is thoroughly oxidized to CO_2_. However, in the natural environment, there are several steps to convert CH_4_ to CO_2_. CH_4_ may firstly be oxidized to methanol, and then formaldehyde, etc. If CH_4_ was not thoroughly oxidized to CO_2_, although it was oxidized, the O_2_ consumption would be low, and there would be less—or even no—CO_2_ generated. This speculation is consistent with the calculated results: CH_4_ generating rates > (O_2_ consuming + CH_4_ escaping) rates > (CO_2_ generating + CH_4_ escaping) rates. Although the methanogenesis fluxes were different in different blocks, the CO_2_ generating flux was almost the same. Methanogenesis may not be the rate-limiting process for the mineralization of hydrocarbons due to a suite of environmental constraints [28,29], although methanogenesis has been deemed to be the rate-limiting process for NSZD [11].

Another possible reason is the overestimated gradients of CH_4_. In the estimated area, besides diffusion, there may be the presence of CH_4_ oxidation and/or methanogenesis, which would vary the diffusion gradients. If the net consumed CH_4_ (the oxidized CH_4_ minus generated CH_4_) in the upper soils was greater than in the deeper soils, the gradient would be overestimated and vice versa. However, it was hard to calculate the net consumed CH_4_ dependent upon the monitored soil gas concentrations. Functional gene quantification may help to differentiate the CH_4_ oxidation or methanogenesis processes.

The significantly negative correlation between the *mcr*A gene and oxygen concentrations was consistent with the consensus that anaerobic conditions are necessary for methanogenesis [30]. Although the *mcr*A gene was expected to be positively correlated with CH_4_, it was not significantly correlated with VOCs. Considering the low VOCs concentrations, therefore, most of the methane might not be generated by the biodegradation of VOCs in the vadose zone but by the biodegradation of petroleum hydrocarbons in the LNAPLs zone, consistent with the hypothesis of the calculation model (Equation (7)). It should be noted that MCR complexes (*mcr*ABG subunits) can also serve as a core gene for anaerobic methane oxidation, but only in a few methanogens [31].

As the *pmo*A product is an O_2_-dependent enzyme [32], the negative correlation with O_2_ suggested that O_2_ was not a limiting factor for methane oxidation at the study depth. The positive correlation with CH_4_ suggested that the amount of CH_4_ provided methane oxidation activity. The *pmo*A concentrations in group III were higher, indicating that the CH_4_ oxidation rates in the deeper soil layers were greater than those in the upper layers. The results also indicated that the CH_4_ oxidation zone occurred in the deeper soils, and the CH_4_ concentration gradients in the shallow depth (<60 cm) were primarily formed by diffusion, which conformed to the application condition of the NSZD assessment model.

Furthermore, *pmo*A abundance was several orders of magnitude higher than that of the *mcr*A gene. This finding suggested that methane at the study depth might be mostly consumed rather than generated by functional microorganisms, and the net consumed CH_4_ in upper soils might be less than those in the lower soils. This would cause an underestimation of the gradients of CH_4_. Therefore, the second possible reason for the greater methanogenesis rates is invalid, and the estimated rates of methanogenesis above were conservative. Therefore, methanogenesis was the rate-limiting process for NSZD at the study site, and the actual NSZD rates roughly equaled the methanogenesis rates, which is consistent with the previous study [11].

### 4.2. Environmental Implications

The calculated NSZD rates differed using different gas gradients. The different rates pointed to the different physicochemical pathways. In this study, the methanogenesis rates might equal the total NSZD rates. The NSZD rate values of 4~40 kg/a.m^2^ are greater than 90% of the 40 LNAPL sites with a 90th percentile value of 4 kg/a.m^2^ [9]. As previously investigated, the greatest NSZD rate was about 12 kg/a.m^2^, which was gained from accidental releases of denatured fuel-grade ethanol-affected sites [33]. The higher NSZD rates in our site might be caused by ongoing leaking from the storage tanks. The generated CH_4_ was almost oxidized beneath 50cm, 40cm, and 30 cm, respectively, in blocks A, B, and C, but more than half of the CH_4_ was not thoroughly converted to CO_2_. Therefore, using the O_2_ consuming or CO_2_ generating rate to evaluate the NSZD as by some previous studies conducted would underestimate the NSZD rate when there was excessive generated CH_4_ that failed to be thoroughly oxidized to CO_2_.

On the other hand, even though the measured blocks were closely located, the NSZD rates varied. This could be caused by the unevenly distributed contaminants, which is commonly found at petroleum-contaminated sites [34]. Therefore, to gain a more accurate NSZD rate, around the source zone, measurements should be carried out as much as possible.

Multiple gases concentrations measurement combined with functional gene qualification would be a powerful means for distinguishing the different physicochemical pathways in NSZD. In the present study, only shallow soil gases and nucleic acid information were collected. Gas concentrations measured at different depths may produce different gradient values. However, the gained gradients are always in the same order of magnitudes [4]. Therefore, using the shallow subsurface gas concentration gradient to assess NSZD is theoretically acceptable. The same methods can also be applied to other petroleum-contaminated sites such as oil fields, as well as petrochemical plants. To verify the proposed deductions and gain more knowledge on NSZD, deeper samples and other molecular biological analysis should be carried out in further studies.

## 5. Conclusions

In this study, NSZD in the vadose zone at a petroleum-hydrocarbon-contaminated site of a purification plant in Northwest China was assessed using the gradient method based on a proposed minimally invasive measurement of multiple gas concentrations. Combined with the quantification results of the *pmo*A and *mcr*A genes, the concentration of CH_4_ was deduced as it was generated from the deep LNAPL-contaminated zone, and the methanogenesis rates were estimated to be 4 to 40 kg/a.m^2^, which can be deemed to represent the NSZD rates. The NSZD rates were greater than those at most of the previously studied sites. The high NSZD rate at the site may be caused by ongoing leaking from the storage tanks. The generated CH_4_ was almost oxidized before escaping into the air, but more than half of the CH_4_ was not thoroughly converted to CO_2_. This was different during NSZD in the middle- or late-evolution stage of the LNAPL release. Therefore, our study added new data to the NSZD rate database and updated current knowledge about NSZD, especially in China.

## Figures and Tables

**Table 1:** The calculated soil gas gradients.

Soil-Gas			O_2_	CO_2_	CH_4_	VOCs	CH_4_
A	Depth (cm)		0–50	0–50	0–50	0–50	50–90
	Gradient ((g/m^3^)/m)	Value	−199.6	149.1	8.0	8.4 × 10^−4^	62.4
		Standard error	22.7	18.8	3.2	6.0 × 10^−3^	19.4
	Adj. R^2^		0.959	0.948	0.583	−0.311	0.757
B	Depth (cm)		0–40	0–40	0–40	0–40	40–90
	Gradient ((g/m^3^)/m)	Value	−179.3	128.9	4.2	1.9 × 10^−2^	87.3
		Standard error	36.4	21.5	2.2	6.2 × 10^−3^	22.6
	Adj. R^2^		0.886	0.921	0.473	0.74	0.736
C	Depth (cm)		0–30	0–30	0–30	0–30	30–60
	Gradient ((g/m^3^)/m)	Value	−633.4	153.9	3.0	1.9 × 10^−3^	1042.0
		Standard error	22.0	16.1	0.4	3.3 × 10^−3^	16.0
	Adj. R^2^		0.998	0.978	0.965	−0.525	0.983
Background	Depth (cm)		0–45	0–45	0–45	0–45	
	Gradient ((g/m^3^)/m)	Value	−6.3	4.1	0.0	0.0	
		Standard error	−1.4	0.0	0.0	0.0	
	Adj. R^2^		0.688	0.994	1	1	

## Data Availability

The data presented in this study are available on request from the corresponding author.
